# Preterm birth and its associated factors in Ethiopia: a systematic review and meta-analysis

**DOI:** 10.4314/ahs.v21i3.43

**Published:** 2021-09

**Authors:** Fikadu Waltengus Sendeku, Fentahun Yenealem Beyene, Azimeraw Arega Tesfu, Simachew Animen Bante, Getnet Gedefaw Azeze

**Affiliations:** 1 Department of Midwifery, College of Medicine and Health Sciences, Bahir Dar University, Bahir Dar, Ethiopia; 2 Department of Midwifery, College of Health Sciences, Woldia University, Woldia, Ethiopia

**Keywords:** Prevalence, pre-term birth, determinants, systematic review, meta-analysis, Ethiopia

## Abstract

**Background:**

Preterm birth is a public health concern globally. In low- and middle-income countries, like Ethiopia, preterm birth is under reported and underestimated. Therefore, this systematic review and meta-analysis assessed the pooled prevalence and associated risk factors for preterm birth in Ethiopia.

**Methods:**

In this review the databases used were PubMed, Google scholar, EMBASE, HINARI and African journal online. Publication bias was checked using a funnel plot and Eggers test.

**Results:**

A total of 30 studies were included in this systematic review and meta-analysis. The overall pooled prevalence of preterm birth in Ethiopia was 11.4% (95% CI; 9.04, 13.76). On pooled analysis, preterm birth was associated with pregnancy-induced hypertension being HIV-positive, premature rupture of membrane, rural residence, the mother having a history of abortion, multiple pregnancies, and anemia during pregnancy.

**Conclusion:**

The national prevalence of preterm birth in Ethiopia was low. Early identifying those pregnant women who are at risk of the above determinants and proving quality healthcare and counsel them how to prevent preterm births, which decrease the rate of preterm birth and its consequences. So, both governmental and non-governmental health sectors work on the minimization of these risk factors.

## Background

According to WHO definition, preterm birth is a birth that occurs before 37 completed weeks of conception or fewer than 259 days from the first date of a woman's last menstrual period for singleton pregnancy. Based on gestational age, it is classified as extremely preterm (<28 weeks), very preterm (28 to < 32 wks.), and moderate to (32 to < 34 weeks) and late preterm (34 to 37 weeks). In other ways on the basis of birth weight, preterm can be classified as low birth weight (1500gm to <2500gm), very low birth weight (<1500 to 1000gm) and extremely very low birth (<1000gm). In addition, on the basis of initiation of labor preterm birth can be categorized into two spontaneous or induced. Spontaneous preterm birth occurs when a pregnant mother goes into labor without the use of drugs or techniques to induce labor before 37 weeks of gestation. Induced preterm birth is a delivery involving labor induction, where drugs or manual techniques are used to initiate the process of labor before 37 weeks of gestation for maternal and fetal indications^1^.

Globally, different studies reported that more than 15 million (11%) babies are estimated to be PTB each year and about 12 million (more than 81%) of these PTB occur in Sub-Sahara Africa and South Asia. Besides, the burden of Pre-term birth ranges between 5% and 18% in the world. In the lower and middle -income countries, on average, 12% of babies are born premature compared with 9% in higher-income countries^2^–^4^.

Studies conducted across the world identified risk factors associated with preterm birth, such as having a history preterm birth, short cervical length, smoking, chronic cough, Short inter-pregnancies interval, anemia, urinary tract infection, certain pregnancy-related complications (such as multiple-pregnancy, pregnancy-induced hypertension, vaginal bleeding, PROM, IUFD, IUGR, polyhydramnios, Oligohydramnious, congenital anomalies of the fetus), lack of antenatal care follow-ups, lifestyle factors (such as low pre-pregnancy weight, and substance use during pregnancy^5^–^8^.

Preterm babies can suffer lifelong effects such as cerebral palsy, mental retardation, visual and hearing impairments, poor health, and growth. Their developmental milestones are negatively affected. Preterm babies require prolonged hospital stay after delivery, repeated hospital admissions in the first year of life and increased risk of acute/chronic lung disease and putting their parents in social and financial problems^1^, ^7^.

## Methods

### Reporting

The report was written by using Preferred Reporting Items for Systematic Reviews and Meta-Analyses (PRISMA)) guideline^9^.

### Searching Strategy and Information sources

We have searched the following finding Items for this review (PRISMA) which was strictly followed by systematic review and meta-analysis guidelines. The databases used were PubMed, Google scholar, EMBASE, HINARI AJOL (African journal online). There were no restriction articles based on publication period. Searching engines were based on adapted PICO principles to search through the above-listed databases to access all the essential articles. To conduct a search of the literature databases, we have used Boolean logic, connectors “OR,” “AND” in combinations^10^. The search strategy for the PubMed database was done as following: Magnitude of preterm birth “OR” prevalence of preterm birth” OR” determinants of preterm birth” OR “ risk factors of preterm birth “OR “magnitude of premature birth “OR “prevalence of premature birth”(MeSH terms) AND birth “OR” parturition “OR” newborn (MeSH terms) “OR” infant AND Ethiopia AND “April 2009(PDat)-April 2020(PDat)”, stated below (Table 1)

**Table 1:** Search for MEDLINE/PubMed and Google Scholar databases to assess preterm birth in Ethiopia

Databases	Searching terms	Number of studies
MEDLINE/PubMed	“preterm birth” OR “premature birth” AND “determinants” OR “predictors” OR “risk factors” OR “associated factors”	303
Google scholar	“preterm birth” or “premature birth” and “determinants” or “associated factors” or risk factors” and “Ethiopia”	30
From other databases	From hand searching using back and front searching and EMBASE	207
Total retrieved articles		540
Number of included studies		30

### Inclusion and exclusion criteria

Cross-sectional, cohort, and case-control studies were included. Articles included in this review were reported the prevalence, or magnitude and associated factors, or determining factors among mothers who were giving birth. Articles were included that were published only in English language literature, published from 2009–2020. Articles without full text and inaccessibility of abstract, commentaries, letters, duplicated studies, anonymous reports, and editorials were excluded.

### Data extraction and Risk of bias

Findings from all databases were exported to Microsoft Excel spreadsheet. Two reviewers (FW, GG) independently extracted the data and reviewed the screened articles. Differences were recon reviewers (FY, SA &AA). Finally, the consensus was reached through a discussion between reviewers. Newcastle-Ottawa Quality Assessment Scale (NOS) for cross-sectional, cohort, and case-control studies were used to assess the methodological quality of a study and to determine the extent to which a study addressed the possibility of bias in its design, conduct, and analysis. All reviewers independently assessed the articles which were included in the review. The average mean score for the cross-sectional studies was 8.91 out of 11, for case-control studies 10.66 out of 22 and Cohort study 14 out of 22. No study that scored below the cutoff point was excluded from the review. All of the included articles scored (NOS) 7 and more can be considered a “good” study and have a low risk of bias for cross-sectional studies and 9 or more scores for case-control and cohort chosen to indicate a high standard for comparative observational studies, stated (S1 Table given below, S2 Table and S3 Table). The last search date was Aprl 15,2020.

### Data collection process

Two independent reviewers (FW, GG) extracted data by using structured data extraction form. The name of the first author, year of study, year of publication, study of region, study area, study design, sample size, the prevalence rate, determinants of preterm birth, and AOR (95% CI) were extracted.

### Outcome of measurement

The measurement outcome of this study had two main outcome variables. The prevalence of preterm birth was the primary outcome of the study, whereas associated factors/determinant for preterm birth were the second outcome variable. The odds ratio was calculated for the common factors of the reported studies. The most common associated factors included in this systematic review and meta-analysis were pregnancy-induced hypertension, being HIV-positive, premature rupture of membrane, rural residence, mother having a history of abortion, anemia during pregnancy and multiple pregnancy.

### Publication bias and heterogeneity

Heterogeneity was checked using I2 and its corresponding p-value. A value of 25%, 50%, and 75% was used to state the heterogeneity test as low, moderate and marked heterogeneity, respectively^11^. The random effect model of analysis was used with the evidence of heterogeneity. Funnel plot and Egger regression test was used to check the existence of publication bias. Sub-group analysis, trim fill and sensitivity analysis were employed to select the most influential risk factors and avoid evidence of publication bias.

### Data analysis

Stata 11 software with forest plots were used to report the estimated pooled prevalence and determinants of each study with the 95% confidence interval (CI). We have conducted subgroup analysis by sample size of participants and year of publication of study due to marked heterogeneity I2 =96.3%. We have also conducted Trim fill and sensitivity analysis to see the effects of a single study on the prevalence of preterm birth. Finally, the odds ratio with 95% CI of pregnancy induced hypertension, being HIV-positive, premature rupture of membrane, rural residence, mother having a history of abortion, anemia during pregnancy and multiple pregnancy were computed.

## Results

### Description of eligible studies

**Figure 1:**
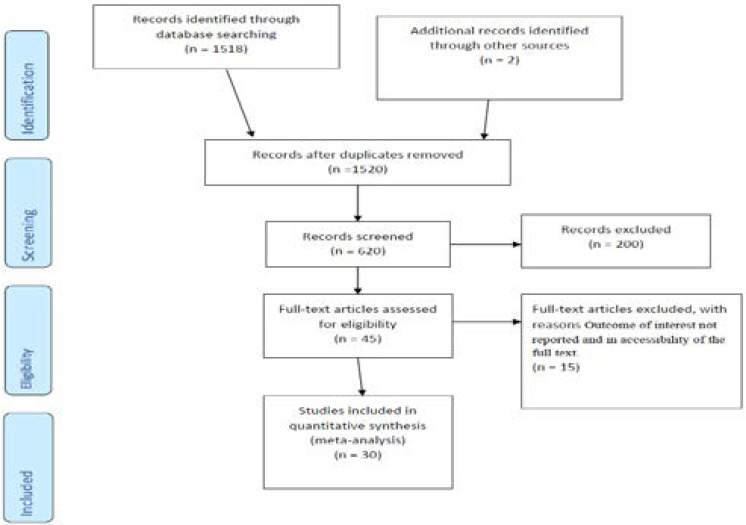
Flow chart of study selection for systematic review and meta-analysis of preterm birth in Ethiopia.

A total of 540 articles were retrieved related to preterm birth through electronic searches. Of those retrieved, 303 papers were from PubMed/MIDLINE, 30 from Google scholars, and 207 from other sources. From the total papers, 88 duplicate and 400 non-eligible papers were identified and excluded during the screening of the titles and abstracts. The remaining 42 articles were given full test review, resulting in 30 papers being considered appropriate and eligible for analysis. Twelve articles were excluded based on the exclusion criteria stated below (Fig. 1).

### Characteristics of the included studies

As a result, 30 studies were met the inclusion criteria to undergo the final systematic review and Meta-analysis. This systematic review and Meta-analysis consist of 23 cross-sectional, 6 case-control and 1 cohort studies with total 17,403 study participants in different regions in Ethiopia (Table 2).

**Table 2:** Study characteristics included in the systematic review and meta-analysis in Ethiopia (n = 30)

Authors	Year of study	Region	Study Design	Sample size	Quality status
Abebe T et al. (35)	2016	Addis Ababa	Cross-sectional	384	Low risk
Abebayehu M et al. (36)	2018	Amhara	case-control	405	Low risk
Muluken D et al. (37)	2017	Amhara	case-control	417	Low risk
Gebrekiros A et al. (38)	2018	Tigray	Cross-sectional	472	Low risk
Dawit G et al (39)	2016	Amhara	Cross-sectional	548	Low risk
Demelash W et al(40)	2017	Oromia	Cross-sectional	325	Low risk
Kahsay G et al(41)	2016	Amhara	Cross-sectional	540	Low risk
Bekele I et al(42)	2015	Oromia	Cross-sectional	220	Low risk
Hayelom G et al(43)	2014	Tigray	Cohort	1152	Low risk
Bayew K et al(44)	2018	Tigray	Cross-sectional	325	Low risk
Tesfaye B et al(45)	2018	Tigray	Cross-sectional	413	Low risk
Girmay T et al(46)	2017/2018	Tigray	case-control	264	Low risk
Samuel D et al(47)	2018	SNNPR	case-control	280	Low risk
Melkamu B et al(48)	2014–2016	Oromia	Cross-sectional	1400	Low risk
Sheka Shemisi et al(49)	2017	0romia	case-control	656	Low risk
Tigist B et al(50)	2013	Amhara	Cross-sectional	422	Low risk
Ayenew E et al(51)	2018	Amhara	Cross-sectional	325	Low risk
Akilew A et al(52)	2013	Amhara	Cross-sectional	481	Low risk
Abdo et al(53)	2015	SNNPR	Cross-sectional	327	Low risk
Abera H et al(54)	2015–2016	Tigray	Cross-sectional	425	Low risk
Cherie N et al (55)	2017	Amhara	Cross-sectional	462	Low risk
Tsegaye and Kassa(56)	2017	SNNPR	Cross-sectional	589	Low risk
Abdo RA et al(57)	2019	SNNPR	Cross-sectional	313	Low risk
Abebe E et al(58)	2012–2013	Amhara	Cross-sectional	3003	Low risk
Tsegaye L et al (59)	2017	SNNPR	Cross-sectional	718	Low risk
Getachew M et al(60)	2018	Amhara	Cross-sectional	1134	Low risk
Eshete A et al(61)	2009	Amhara	Cross-sectional	295	Low risk
Hailemariam Workie(62)	2015	Tigray	case-control	340	Low risk
Eskeziaw A et al(63)	2017	Amhara	Cross-sectional	462	Low risk

### Prevalence of preterm birth among mothers who gave birth at health institutions in Ethiopia

**Figure 2:**
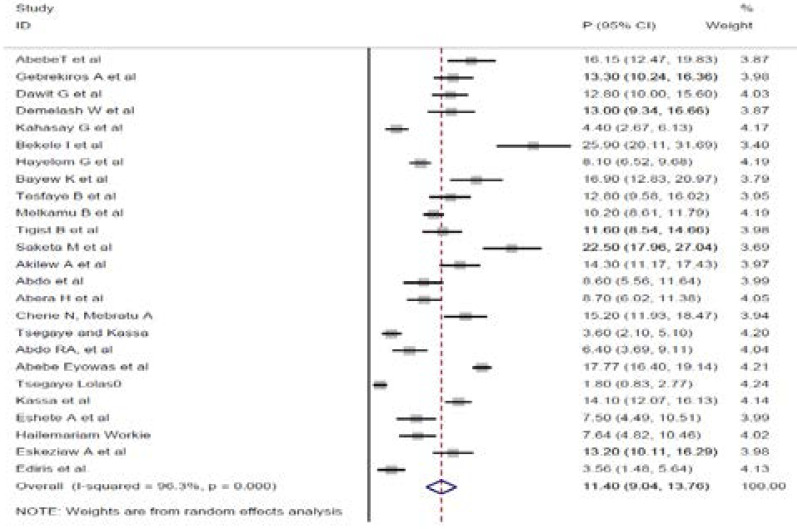
Forest funnel plot of the pooled prevalence of preterm birth in Ethiopia

The overall pooled prevalence of preterm birth in Ethiopia is presented with a forest plot (Fig. 2). Therefore, the pooled estimated prevalence of preterm birth in Ethiopia was 11.4% (95% CI; 9.04, 13.76; I2=96.3%, P≤0.001).

### Subgroup analysis

Subgroup analysis was done with the evidence of heterogeneity. Hence, the Cochrane I2 statistic =96.3%, P≤0.001) with evidence of marked heterogeneity. Therefore, subgroup analysis was conducted using the sample size of participants and year of publication of the articles. Based on the subgroup analysis, the prevalence of preterm birth in Ethiopia was 10.73% (95%CI: 7.67,13.79) I2=97.3%, P≤0.001) in which sample size was≥400 (Table 3).

**Table 3:** Subgroup pooled prevalence of preterm birth among mothers who gave birth at health institutions in Ethiopia (n= 25)

Variables	Subgroup	No of studies	Prevalence% (95%CI)	I^2^ (%)	P-value
Sample size	≥400	15	10.73(7.67,13.79)	97.3	≤0.001
	<400	10	12.48(8.56,16.40)	93.1	≤0.001
Year of publication	2014–2018	16	10.98(8.12,13.85)	95.7)	≤0.001
	≥2019	9	12.15(7.74,16.57)	97.2	≤0.001

### Publication bias

A funnel plot was assessed for the asymmetry distribution of preterm birth among mothers who gave birth at health institutions in Ethiopia by visual inspection. Egger's regression test showed with a p-value of 0.003 indicated for the existence of publication bias. Hence, trim and fill analysis was conducted to overcome the publication bias. Eleven studies filled with 25 studies and overall, 36 studies were enrolled and computed through the trim and fill analysis with a pooled prevalence of 6.52% (95% CI; 3.97–9.07) using a random effect model (Fig. 3 a & b).

**Figure 3:**
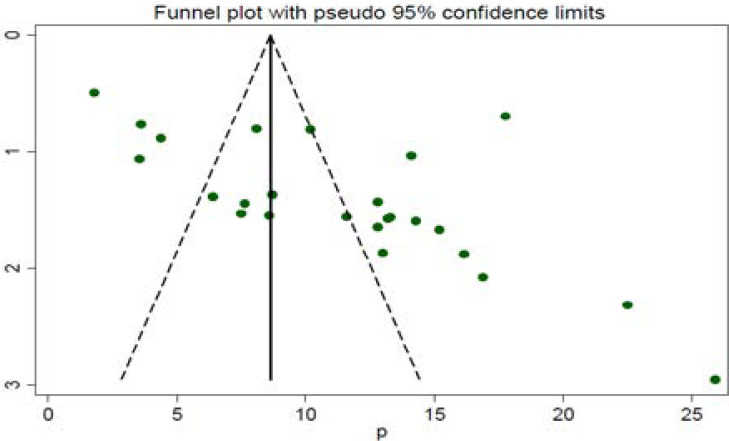
Funnel plot of publication bias a (before an adjustment

**Figure 3b:**
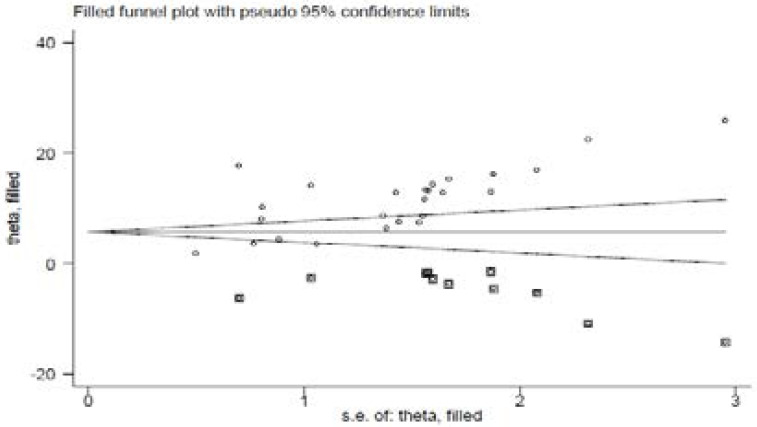
(after trim-fill analysis was computed)

### Sensitivity analysis

**Figure 4:**
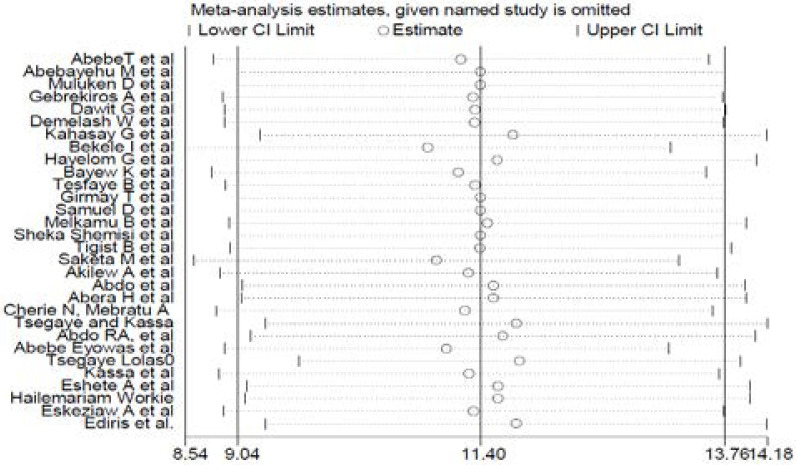
Sensitivity analysis of the pooled prevalence of preterm birth in Ethiopia

This review showed that the point estimate of its omitted analysis lies under the confidence interval of the combined analysis (Fig 4).

Risk factors associated with preterm birth among mothers who gave birth at health institutions in Ethiopia

The most common associated factors included in this systematic review and meta-analysis were pregnancy-induced hypertension, being HIV-positive, premature rupture of membrane, rural residence, mother having a history of abortion, multiple pregnancy, and anemia during pregnancy.

Women who have pregnancy-induced hypertension (AOR: 5.11, 95%CI: 3.73, 7.01)) was positively associated with preterm birth. No heterogeneity (I2=0.0%; p-value=0.872) was detected among the included studies; due this reason, the fixed effect model was calculated. Moreover, the possibility of publication bias was not detected using Egger's tests with a p-value of 0.568.

Women who is HIV-positive were the predictors of preterm birth (AOR: 4.74; 95%CI: 2.79, 8.05). No heterogeneity (I2=0.0%; p-value=0.629) was detected among the included studies; for this reason, the fixed effect meta-analysis model was computed. Moreover, no possibility of publication bias was detected using Egger's tests with a p-value of 0.595.

We found that women who had premature rupture of membrane four times (AOR: 5.36, 95%CI: 3.76, 7.64)) greater at increased risk the likelihood of having a preterm birth compared to their counterparts. Moderate heterogeneity (I2=56.8%; p-value=0.031) was detected among the studies. Therefore, random effect model meta-analysis was employed. Furthermore, no possibility of publication bias was detected using Egger's tests with a p-value of 0.762.

Being a rural residency was one the significant risk factor for preterm birth (AOR: 2.35, 95%CI: 1.56, 3.55). No heterogeneity (I2=0.0%; p-value=0.839) was detected among the included studies; for this reason, the Fixed effect meta-analysis model was computed. Moreover, no existence of publication bias was declared d using the Egger's tests with a p-value of 0.314.

Pregnant women who were anemic (AOR: 3.41, 95%CI: 2.1, 5.56)) were positively associated with preterm birth. Low heterogeneity (I2=26.9%; p-value=0.251) was detected among the included studies; for this reason, the random effect meta-analysis model was computed. Furthermore, publication bias was not detected using Egger's tests with a p-value of 0.657.

Pregnant women who have two or more fetus in intrauterine (AOR: 3.60 95%CI:2.49, 5.19) were positively associated with preterm birth. No heterogeneity (I2=0.0%; p-value=0.429) was detected among the included studies; for this reason, the fixed effect meta-analysis model was computed. Furthermore, publication bias was not detected using Egger's tests with a p-value of 0.835.

Women having a history of abortion (AOR: 2.92, 95%CI: 1.91, 4.47) were the determining factor of preterm birth. No heterogeneity (I2=0.0%; p-value=0.726) was detected. Hence, the fixed effect meta-analysis model was computed. Furthermore, the existence of publication bias was not detected using Egger's tests with a p-value of 0.835 (Table. 4).

**Table 4:** Summary of associated risk factors with preterm birth in Ethiopia

Variables	Model	Egger test (P-value)	Status of heterogeneity	AOR (95%CI)	I^2^ (%)	P-value
PIH	Fixed	0.568	No heterogeneity	5.11(3.73, 7.01)	0.0	0.872
HIV-Positive	Fixed	0.595	No heterogeneity	4.74(2.79, 8.05)	0.0	0.629
PROM	Random	0.762	Moderate heterogeneity	5.36(3.76, 7.64)	56.8	0.031
Rural residence	Fixed	0.314	No heterogeneity	2.35(1.56, 3.55)	0.0	0.839
Hx of abortion	Fixed	0.835	No heterogeneity	2.92(1.91, 4.47)	0.0	0.726
Multiple pregnancy	Fixed	0.835	No heterogeneity	3.60(2.49, 5.19)	0.0	0.429
Anemia during pregnancy	Random	0.657	Low heterogeneity	3.41(2.1, 5.56)	26.9	0.251

## Discussion

In this meta-analysis, the overall pooled prevalence of preterm birth among mothers who gave birth at health institutions in Ethiopia was 11.4% (95% CI; 9.04,13.76). This finding agrees with studies conducted in Tanzania 13% 12, India 12.95% 13 and Brazil 13.7% 14. The possible explanation might be due to all the above countries might be implementing the recommendation of comprehensive health-care system for pregnant women.

The finding of this review and meta-analysis was lower than the study done in India 15% 15, Brazil 21.7% 16, Kenya 18.3% 8 and 20.2% 8, India 28.25% 17 and Nigeria 24% 18. This difference could be due to in our country setting having lower risk factors for preterm birth as compared to those countries. For example, in Nigeria, there is the highest rate of multiple gestations. Multiple gestation causes over distended uterus, and can precipitate to preterm birth, and multiple gestations are a known predisposing factor for preterm birth.

The finding of this review was higher than the studies conducted in Kenya 8.3%, two different studies in Iran 6.3% 19 and 8.4% 20 and the United Arab Emires 6.3%^21^. The possible justification might be due to our country setting having higher risk factors and having lower quality care for pregnant mother during pregnancy, even though there is identified known risk factors for preterm birth as compared to those countries.

The study determined that women who had developed pregnancy-induced hypertension were 5.11-time (AOR: 5.11, 95%CI: 3.73, 7.01)) greater risk with preterm birth than the counterpart. The finding is consistent with the study done in East Africa 22, China^23^, India 17, Kenya (24), Nigeria 25, Brazil 26, and two different studies in Iran 19,20. The possible explanation might be due to the existing scientific evidence which speculates that Pregnancy induced hypertension is linked to vascular and placental damage, which in turn reduces placental blood flow and leads to uteroplacental insufficiency resulting in obstetric emergencies that require termination of pregnancy as a lifesaving for both the mother and fetus. Abruptio placenta is another complication of pregnancy-induced hypertension that may require termination of pregnancies 27.

This review showed that HIV-positive pregnant mothers were 4.7 times (AOR: 4.74, 95%CI: 2.79, 8.05) more at increased risk of having a preterm birth than negative mothers. This is in agreement with a study conducted in South Africa 28 and Tanzania 29. It could be explained by the effect of ART drugs and have low immunity status of the mother at greater risk for preterm birth.

We found that women who had premature rupture of membrane four times (AOR: 5.36, 95%CI: 3.76, 7.64)) greater at increased risk the likelihood of having preterm birth as compared to their counterparts. This is in agreement with a study done in two different studies in Iran 19,20. Kenya^24^, Nigeria 25 and India 17. This might be due to the fact that endogenous prostaglandins released after ruptured membrane that initiates the uterine contraction, thereby cause preterm birth.

Pregnant women who are living in rural areas 2.4 times (AOR: 2.35, 95%CI: 1.56, 3.55) more at increased risk for preterm birth than in urban areas, which is consistent with a study conducted in Kenya^8^. This is the fact that pregnant women who are living in rural areas suffer from different risk conditions like face-unbalanced diet, walk a long distance for fulfilling their family's needs, do extraneous work, and have poor access health care system, thereby distance from health facility that might contribute to preterm birth.

Pregnant women who were anemic (AOR: 3.41, 95%CI: 2.1, 5.56)) were positively associated with preterm birth. This is in agreement a study done in India 17, Malawi 30, China 31 and East Africa 22. This is because of the biological mechanisms of anemia, iron deficiency or both could cause preterm delivery. In fact, anemia and iron deficiency can induce stress and maternal infections, which in turn stimulate the synthesis of Corticotrophin-Releasing Hormone (CRH) that elevated CRH concentrations to be a known risk factor for preterm birth 32.

This review showed that multiple gestation has been associated with the increased likelihood of preterm birth (AOR: 3.60 95%CI:2.49, 5.19). This finding agrees with those studies done in Kenya 8, India 17, Iran 20. This might be due to multiple gestation is associated with uterine over-distension, which causes increased gap junction of myometrial muscles and induce the oxytocin receptors. Finally, it can initiate uterine contraction that is resulting preterm birth 33.

Another risk factor associated with preterm birth had a history of abortion (AOR: 2.92, 95%CI: 1.91, 4.47)), which is consistent those studies done in Brazil 34, Iran^17^ and Tanzania 12. This is because during surgical evacuation of the uterus mechanically stretches the cervix could cause cervical incompetency, which in turn predispose preterm birth for subsequent pregnancies.

## Strength

This review seems to be done first in Ethiopia. In addition, the included studies in this review were cross-sectional, case-control, and cohort show temporal cause effect relationships due to its nature of the design.

## Limitation

This review has been limited to articles published only in English languages that cause reporting bias. Data were not found in all regions of the country this can cause representative problems.

## Conclusion

The national prevalence of preterm birth in Ethiopia was low. The most common associated factors included in this systematic review and meta-analysis were pregnancy-induced hypertension, being HIV-positive, premature rupture of membrane, rural residence, the mother having a history of abortion, multiple pregnancy, and anemia during pregnancy. This review can be used to base line for health policy makers, clinicians, and program officers to design action plan work for prevention and intervention measures on preterm birth. Early identifying those pregnant women who are at risk of the above determinants and proving quality healthcare and counsel them how to prevent preterm birth which may decrease the rate of preterm birth and its consequences.

## Abbreviations

AOR-Adjusted Odd Ratio, ART-Anti-Retroviral Therapy, CI- Confidence interval, HIV-Human Immuno Deficiency Virus, PIH-Pregnancy Induced Hypertension, PROMPremature Rupture of membrane.

## Declarations

### Availability of data and materials

All related data have been presented within the manuscript.
